# Hepatoprotective Effects of Hyaluronic Acid-Preconditioned Bone Marrow Mesenchymal Stem Cells against Liver Toxicity via the Inhibition of Apoptosis and the Wnt/β-Catenin Signaling Pathway

**DOI:** 10.3390/cells12111526

**Published:** 2023-06-01

**Authors:** Amira Awadalla, Eman T. Hamam, Sally Abdallah Mostafa, Seham Ahmed Mahmoud, Khalid Mohamed Elazab, Ahmed Mohamed El Nakib, Mamdouh Eldesoqui, Mohamed El-Sherbiny, Omar A. Ammar, Rasha Hamed Al-Serwi, Mohamed A. Saleh, Amira Sarhan, Mohamed Ali

**Affiliations:** 1 Center of Excellence for Genome and Cancer Research, Urology and Nephrology Center, Mansoura University, Mansoura 35516, Egypt; 2 Medical Biochemistry and Molecular Biology Department, Faculty of Medicine, Mansoura University, Mansoura 35516, Egypt; 3 Chemistry Department, Faculty of Science, Zagazig University, Zagazig 44519, Egypt; 4 Department of Biology, Faculty of Science, Jazan University, Jazan 82511, Saudi Arabia; 5 Department of Tropical Medicine, Faculty of Medicine, Mansoura University, Mansoura 35516, Egypt; 6 Department of Anatomy, Faculty of Medicine, Mansoura University, Mansoura 35516, Egypt; 7 Department of Basic Medical Sciences, College of Medicine, AlMaarefa University, P.O. Box 71666, Riyadh 11597, Saudi Arabia; 8 Basic Science Department, Delta University for Science and Technology, Gamasa 35712, Egypt; 9 Department of Basic Dental Sciences, College of Dentistry, Princess Nourahbint Abdulrahman University, P.O. Box 84428, Riyadh 11671, Saudi Arabia; 10 Department of Clinical Sciences, College of Medicine, University of Sharjah, Sharjah 27272, United Arab Emirates; mohamed.saleh@sharjah.ac.ae; 11 Research Institute for Medical and Health Sciences, University of Sharjah, Sharjah 27272, United Arab Emirates; 12 Department of Pharmacology and Toxicology, Faculty of Pharmacy, Mansoura University, Mansoura 35516, Egypt; 13 Biochemistry Division, Chemistry Department, Faculty of Science, Zagazig University, Zagazig 44519, Egypt

**Keywords:** hyaluronic acid, BMSCs, hepatotoxicity, Doxorubicin

## Abstract

Background: Doxorubicin (DOX) is widely used to treat a variety of malignancies in both adults and children, including those of the bladder, breast, stomach, and ovaries. Despite this, it has been reported to cause hepatotoxicity. The recent discovery of bone marrow-derived mesenchymal stem cells’ (BMSCs) therapeutic effects in the context of liver diseases suggests that their administration plays a part in the mitigation and rehabilitation of drug-induced toxicities. Objectives: This study investigated whether bone BMSCs could reduce DOX-induced liver damage by blocking the Wnt/β-catenin pathway that causes fibrotic liver. Materials and methods: BMSCs were isolated and treated with hyaluronic acid (HA) for 14 days before injection. Thirty-five mature male SD rats were categorized into four groups; group one (control) rats were supplemented with saline 0.9% for 28 days, group two (DOX) rats were injected with DOX (20 mg/kg), group three (DOX + BMSCs) rats were injected with 2 × 10^6^ BMSCs after 4 days of DOX injection, group four (DOX + BMSCs + HA) rats were injected with 0.1 mL BMSCs pretreated with HA after 4 days of DOX. After 28 days the rats were sacrificed, and blood and liver tissue samples were subjected to biochemical and molecular analysis. Morphological and immunohistochemical observations were also carried out. Results: In terms of liver function and antioxidant findings, cells treated with HA showed considerable improvement compared to the DOX group (*p* < 0.05). Moreover, the expression of inflammatory markers (TGFβ1, iNos), apoptotic markers (Bax, Bcl2), cell tracking markers (SDF1α), fibrotic markers (β-catenin, Wnt7b, FN1, VEGF, and Col-1), and ROS markers (Nrf2, HO-1) was improved in BMSCs conditioned with HA in contrast to BMSCs alone (*p* < 0.05). Conclusion: Our findings proved that BMSCs treated with HA exert their paracrine therapeutic effects via their secretome, suggesting that cell-based regenerative therapies conditioned with HA may be a viable alternative to reduce hepatotoxicity.

## 1. Introduction 

Cancer is among the most common diseases, having caused up to 10 million deaths in 2020, with an annual incidence of 19.3 million cases [1]. Most medications used for systemic cancer treatment are chemotherapeutics that target uncontrolled cancer cell growth and proliferation [2].

Doxorubicin (DOX) is an anthracycline chemotherapeutic drug that was initially derived from *Streptomyces peucetius* [3]. It is often recommended for a range of malignancies, such as acute lymphomas, gastrointestinal, sarcomas, ovarian, bone, and several pediatric cancers [4]. DOX exerts its anti-cancer effects by intercalating into the DNA double helix and interrupting topoisomerase II-mediated DNA repair and by producing free radicals that destroy proteins, cell membranes, and DNA. The latter pathway involves oxidizing DOX to semiquinone, which is subsequently transformed back into DOX by generating reactive oxygen species (ROS) [5].

High dosages and continuous usage of DOX may cause hepatotoxicity, adipose tissue damage, cardiotoxicity, and recurrent and resistant malignancies [6]. DOX-induced liver impairment varies from non-specific alterations in liver structure to acute liver failure, cirrhosis, jaundice, and an increase in serum liver enzyme AST as well as ALT [7]. 

DOX markedly increases the production of intrinsic apoptotic molecules like BCL2-Associated X Protein (Bax) and extrinsic apoptotic molecules like Fas with elevated levels of GPx and SOD antioxidant enzymes while decreasing glutathione (GSH) levels [8]. Consequently, it is of critical importance to create an antioxidant-rich combination therapy with DOX that does not compromise the effectiveness of DOX as an anti-tumor medicine, but rather improves the efficacy of cancer chemotherapy [9].

The Wnt/β-catenin pathway is a conserved, complicated evolutionarily process that affects basic physiologic and pathological functions. Wnt/β-catenin is typically dormant in mature healthy livers, but it can be reactivated when cells are being renewed or regenerated, in addition to reactivating in the context of some pathological disorders, illnesses, pre-malignant dysfunction, and cancer. Research in rodents reveals that Wnt signaling is crucial during liver regeneration for the activation of oval cells, which are precursor cells activated after hepatocyte or cholangiocyte proliferation is disrupted [10].

Mesenchymal stem cells (MSCs) are multipotent with a high capacity for in vitro differentiation and proliferation. Adipose tissue, bone marrow, and the umbilical cord are accessible sources of MSCs. Intravenously given drugs may move to injured tissues and trigger tissue healing via paracrine actions [11]. Bone marrow MSCs (BMSCs) alleviate numerous diseases. Due to differentiation and paracrine actions, BMSCs assist with liver disease therapy. After damage to the liver, BMSCs may become liver epithelial cells. BSMCs merging with liver epithelial cells may help repair and restore liver tissue by lowering oxidative stress and liver cell death [12]. 

The current treatment results from using BMSCs in liver illnesses suggest that MSC injection may have an anti-drug-induced toxicity role. The most crucial aspect of tissue restoration is the migratory mechanism of the injected BMSCs to damaged regions [12]. As a result, techniques for increasing BMSC homing to wounded hepatic tissues must be devised. Hyaluronic acid (HA) is an extracellular matrix and synovial fluid polysaccharide. Hyaluronan influences embryogenesis, tissue regeneration, inflammation, migration, and proliferation [12].

Hyaluronan-induced signaling arises via receptor contacts, although its signal transduction mechanism in cells is unknown. Hyaluronan affects adhesion, proliferation, differentiation, and migration by binding to certain cellular receptors. Moreover, hyaluronan reduces the effects of nitrogen and reactive oxygen species on mtDNA repair, ATP generation, and cell survival [13]. High-molecular-weight hyaluronan has previously been demonstrated to enhance the growth rate at initial passages, lengthen the survival period with a considerable decrease of cellular senescence throughout the subculture, and maintain the differentiation capability of mice adipose-derived stem cells (mADSCs) [14]. However, the molecular pathway through which HA-conditioned BMSCs could protect the liver against chemotherapies is not clear. Therefore, our study was implemented to investigate the effect of HA in the migration of the transplanted cells in DOX-induced hepatotoxicity via reducing apoptosis and blocking the Wnt/β-catenin signaling cascade.

## 2. Materials and Methods

### 2.1. In Vitro Study

#### BMSC Isolation, Expansion, Characterization, and HA Treatment

Rats were sacrificed and the bone marrows were harvested from each bone and flushed through Dulbecco’s modified Eagle media (DMEM) supplemented with 1000 u/mL penicillin. The isolated cells were placed in 25 tissue culture flasks with appropriate media and kept in the incubator at 37 °C and 5% CO_2_. The medium was changed every three days afterward. Then, the cells were subcultured using trypsin/EDTA after reaching 90% confluence [15]. Cells were analyzed using flow cytometry to verify that the BMSCs maintained their phenotypic features in vitro, as they express the markers of BMSCs (CD44 and CD90) with no hematological or endothelial marker expression (CD45 and CD34) [16]. Cells were divided into 2 groups: group 1 was cultured in media without any treatment and group 2 cells were treated with 1 mg/mL HA for 14 days as previously described by La Manna et al. [17]. Real-time PCR was used to confirm the expression level of the HA binding receptor CD44 and SDF1α after HA treatment, and the primer sequence used for CD44 and SDF1α is listed in Table 1.

### 2.2. Assessment of Cell Proliferation and Viability

BMSCs were counted using Kit-8 (CCK-8, Sigma, Barcelona, Spain) to assess proliferation at four different time periods (1, 5, 7, and 14 days) by seeding them at a density of 10 × 10^3^ cells/well in 96-well plates, then each well was treated with CCK-8 reagent and put into a CO_2_ incubator for three hours. Using a microplate spectrophotometer, we determined the optical density at 450 nm (Epoch, Biotek Instruments, Winooski, VT, USA). Similarly, the vitality of the BMSCs and HA-BMSCs was evaluated throughout the period of 14 days in the culture at 0.1, 0.5, 1, and 1.5 mg/mL of HA, respectively. Data were exported to Microsoft Excel and presented as Mean ± SD.

### 2.3. Experimental Animals

Thirty-five mature male Sprague–Dawley rats at an age of 6 weeks weighing 180 ± 200 g were maintained in a controlled environment of 24 °C, with relative humidity between 50 and 70%, and a 12-h light–dark cycle. The rats in the experiment had unlimited access to food and drink. All procedures involving the care and use of laboratory animals conformed to all applicable regulations (ILAR 1996). 

### 2.4. Study Design and Animal Groups

The rats were split across four groups: Control group (n = 5): administered with 0.9% saline; DOX Group (n = 10): received DOX (20 mg/kg) in tail veins on the first day, then saline was administered for the next 28 days; DOX + BMSCs group (n = 10): after 4 days of DOX (20 mg/kg) injection, BMSCs (2 × 10^6^ cells in 0.1 mL DMEM/rat) were administered intravenously through the tail vein; DOX + BMSCs + HA group (n = 10): received the same treatment as the DOX group and were administered with 0.1 mL BMSC cells pretreated with HA after 4 days of DOX (20 mg/kg) injection. Under inhaled general anesthesia, blood samples were taken from the heart before the animal was sacrificed. In addition to that, tissue from the liver was obtained for the purposes of biochemical, structural, and molecular analyses.

### 2.5. Biochemical Assay

The levels of albumin, AST, and ALT were estimated in serum using a colorimetric approach with special kits (Biodiagnostic Co., Giza, Egypt), following the manufacturer’s guidelines.

### 2.6. Evaluation of Oxidant/Antioxidant Parameters

In accordance with the manufacturer’s instructions, reduced glutathione (GSH), malondialdehyde (MDA) contents, and superoxide dismutase (SOD) activity in liver tissues were measured in all groups (Biodiagnostic Co., Giza, Egypt).

### 2.7. Real-Time Polymerase Chain Reaction

Real-time PCR was applied based on the instructions provided by the manufacturer. The Trizol reagent was used to extract total RNAs from liver tissues. Gene expression of the ROS marker (Nrf-2 and HO-1), stem cell trafficking marker (SDF1α), inflammatory markers (iNos, TGFβ1, IL-6, and TNF-α), apoptotic marker (BCL2 and Bax), and fibrotic markers (VEGF, Wnt7b, FN1, Col-1, and β-catenin) were evaluated using the Step One plus for SYBR Green PCR Kit (Qiagen, Germany). The primer sequence was summarized in Table 1. GAPDH was considered to be an endogenous control allowing for the quantification of relative gene expression. The data were calculated using the 2^−∆∆C (T)^ method [18].

### 2.8. Histopathological Studies

The liver tissues were stained with hematoxylin and eosin after fixation with 10% formalin, dehydration with an increasing alcohol series, being washed in xylene twice, and, lastly, being embedded in molten paraffin. Using a rotary microtome, slices were cut to a thickness of 5 mm on sterile slides [19].

### 2.9. Immunohistochemistry

The expression of SDF1α, TGF1, and β-catenin antibodies was observed in the liver tissue of all research groups using immunohistochemical labeling. Paraffin-embedded slices were treated with a 10 mM citrate buffer of pH 6 lasting for 30 min to activate the antigens. Following washing, a secondary biotinylated antibody was kept at RT for an hour; the sections were cleansed again with PBS, and the response color was developed using DAB staining. Before being viewed with an Olympus BX51 light microscope, the cut tissues were counterstained with hematoxylin, dried, and mounted on a coverslip. Five sections were chosen at random from each group, and the intensity of the staining was measured using Image J software (v 1.53, National Institutes of Health, Bethesda, MD, USA).

### 2.10. Statistical Studies

The statistical significance of the differences between the groups was determined using one-way analysis of variance (ANOVA) and least significant difference (LSD) analysis. The results were given as a mean ± standard deviation, and *p* < 0.05 was used for statistical significance.

## 3. Results

### 3.1. In Vitro Treatment of BMSCs with HA

#### Characteristics of BMSCs, Cell Viability, and Proliferation

**Figure 1:**
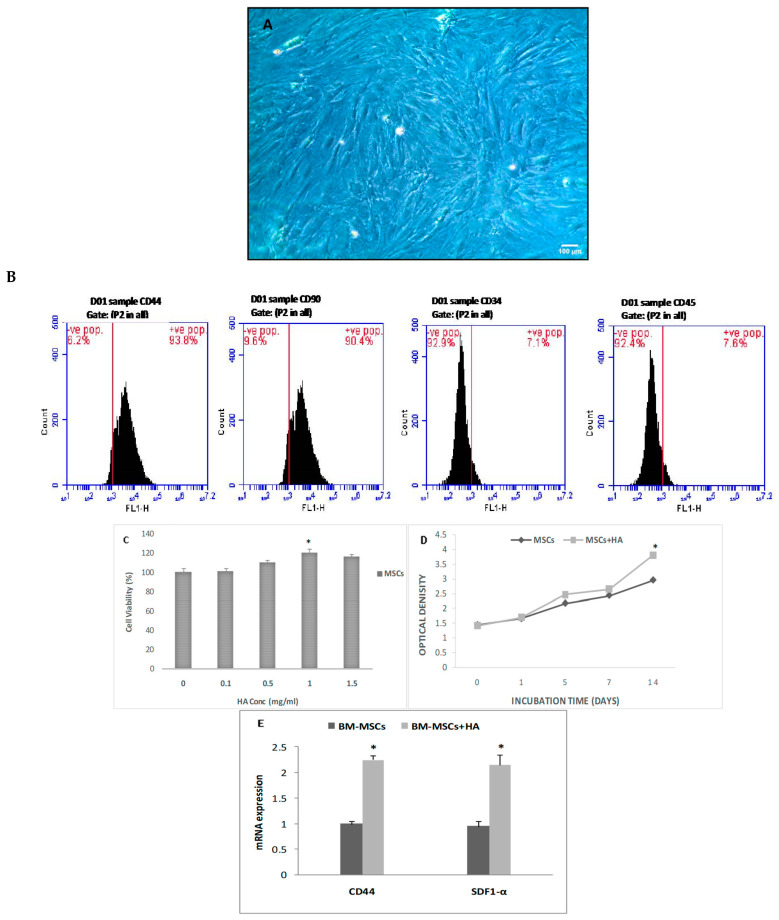
In vitro studies of HA-BMSCs. (**A**) BMSCs treated with HA at passage 3 (magnification 100×), (**B**) flow cytometric analyses for the markers of BMSCs, (**C**) cell viability using different concentrations of HA, (**D**) the effect of HA treatment on the proliferation rate of BMSCs, (**E**) gene expression of CD44 and SDF1α in HA-treated and untreated cells. * *p* < 0.05.

The cell confluence in the BMSCs was between 70 and 80%, and the cells exhibited a long spindle shape, very similar to that of fibroblasts (Figure 1A). Results from a phenotypic study of BMSCs showed that they were positive for the mesenchymal markers CD44 and CD90 (93.8% and 90.4%, respectively) and negative for the hematopoietic markers CD34 and CD45 (92.9 and 92.4%, respectively) (Figure 1B). The viability of the BMSCs at various HA doses demonstrated that cell number increased with increasing HA concentration, with the highest viability at 1 mg/mL (*p* < 0.05; Figure 1C). Moreover, BMSCs expanded in HA-treated media throughout a 14-day period (Figure 1D). Furthermore, HA treatment increased gene expression of CD44 and SDF1α as compared to untreated cells (*p* < 0.05; Figure 1E).

### 3.2. Impact of HA-Treated BMSCs on Liver Function

Liver enzyme levels in the DOX group were significantly higher than those in the control group (*p* < 0.01). There was also a notable decrease in albumin levels in the DOX group compared to the control group. As compared to the DOX group, blood liver enzymes were significantly lower in the BMSCs and HA + BMSCs groups (*p* < 0.01). Moreover, there was a notable increase in albumin levels in the BMSCs and HA + BMSCs groups as compared to the DOX group (*p* < 0.01). The data are shown in Table 2.

### 3.3. Effect of BMSCs on Oxidative Stress in Liver Tissues

The protective efficacy of HA and BMSCs on oxidative stress status was evaluated in all the experimental groups. The results confirmed that the activity of SOD, GSH, and CAT decreased considerably in the DOX group in contrast to the control (*p* < 0.05). It should be noted, however, that SOD, GSH, and CAT activity increased considerably more in the groups that received BMSCs and HA + BMSCs than in the groups that received DOX. Moreover, HA + BMSCs showed better therapeutic efficacy than BMSCs, indicating that HA-treated cells are more successful in reducing oxidative stress (*p* < 0.05). MDA levels were considerably greater in the DOX group when compared to the control group and were significantly lower in both treated groups (BMSCs and HA + BMSCs) when compared to the DOX group (*p* < 0.05). Notably, HA-treated BMSCs diminished the level of MDA more than untreated BMSCs did (Table 2).

### 3.4. Effect of HA-Treated BMSCs on the Expression of Various Genes

**Figure 2:**
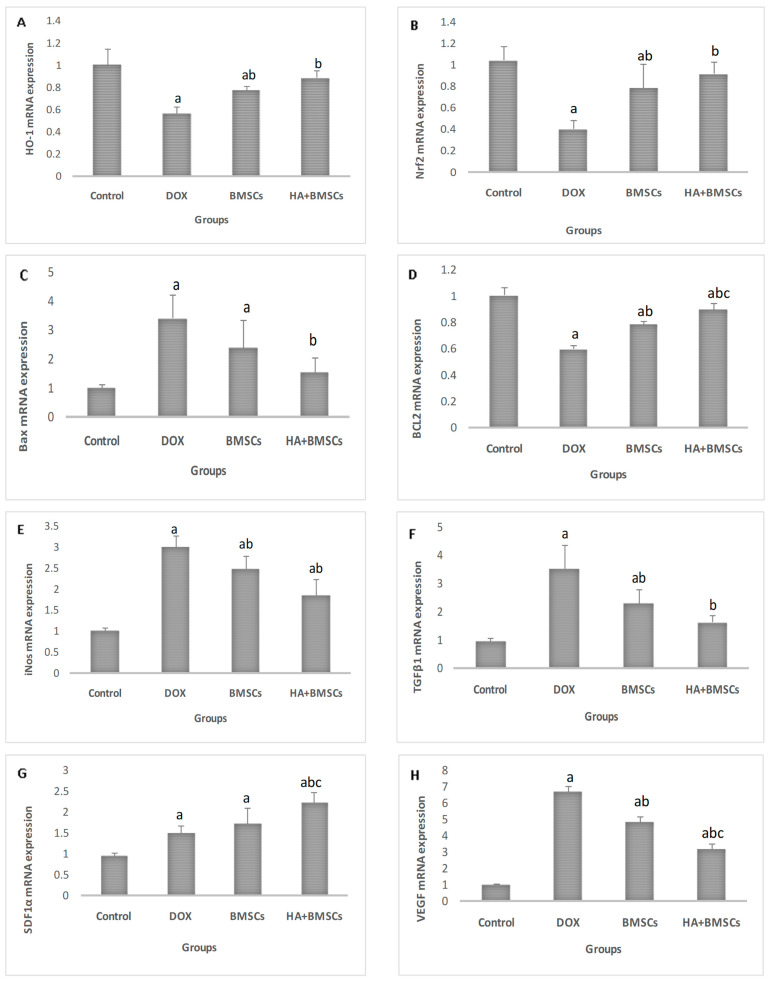

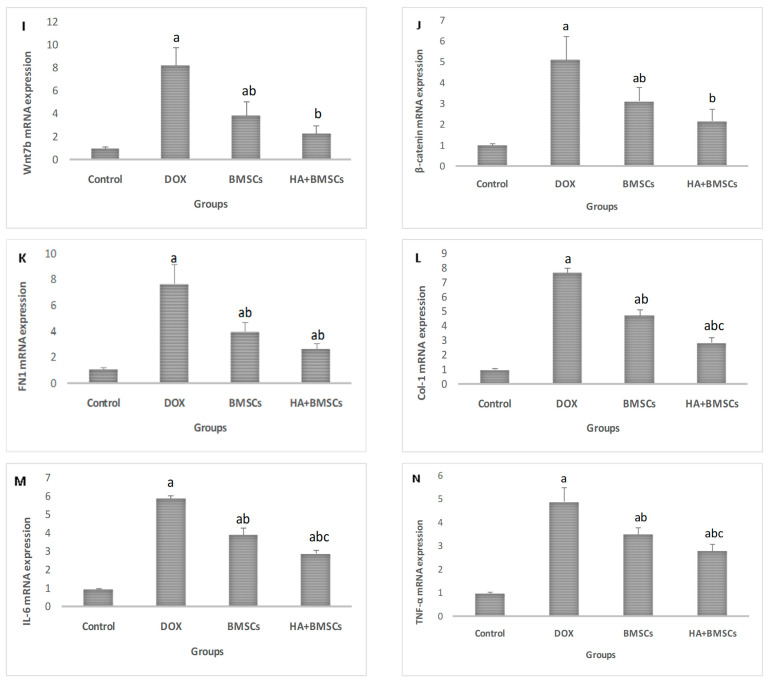
Effect of BMSC and BMSC + HA administration on the gene expression in the control and different treated groups; (**A**) HO-1, (**B**) Nrf2, (**C**) Bax, (**D**) BCL2, (**E**) iNos, (**F**) TGFβ1, (**G**) SDF1α, (**H**) VEGF, (**I**) Wnt7b, (**J**) β-catenin, (**K**) FN1, (**L**) Col-1, (**M**) IL-6, and (**N**) TNF. The data are reported as mean ± SD. Significant difference ^a^ vs. control, ^b^ vs. DOX, and ^c^ vs. BMSCs.

The expression of ROS markers (Nrf-2 and HO-1), stem cell trafficking marker (SDF1α), apoptotic markers (BCL2 and Bax), fibrotic markers (VEGF, Wnt7b, β-catenin, FN1, and Col-1), and inflammatory markers (TGFβ1, iNos, IL-6, and TNF-α) in liver tissues were assessed. The Nrf-2, HO-1, BCL2, and SDF1α expression levels were considerably upregulated in the treated groups in contrast to the DOX group (*p* < 0.01), while the expressions of VEGF, Wnt7b, β-catenin, FN1, Col-1, TGFβ1, iNos, Bax, IL-6, and TNF-α were significantly downregulated in all treated groups in comparison to the DOX group (*p* < 0.01; Figure 2).

### 3.5. Effect of HA-Treated BMSCs on Histopathological Liver Changes

**Figure 3:**
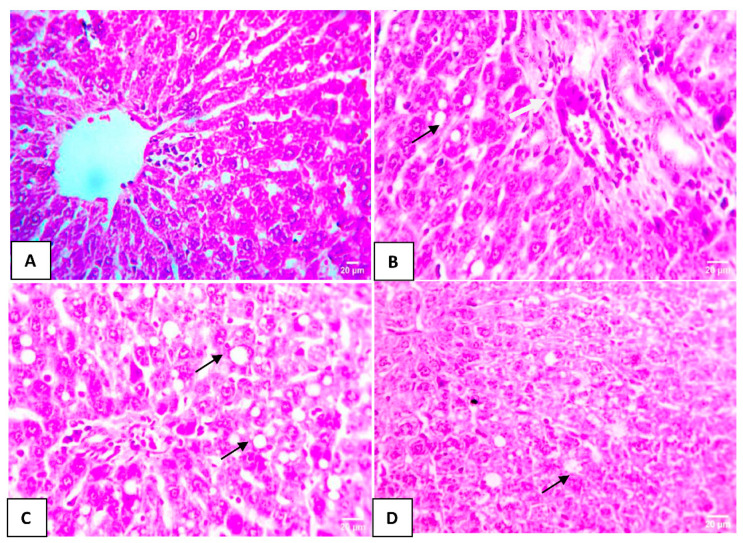
Images of H&E-stained liver slices under the microscope showed normal hepatocytes arranged in cords in the control group (**A**). In liver slices from the DOX group, multiple multifocal lesions of hepatic macrovesicular steatosis in hepatocytes (black arrows) and mild portal fibrosis with a few leukocytic cell infiltrations (white arrow) are seen (**B**). In the DOX + BMSCs group, mild macrovesicular steatosis in a few hepatocytes (black arrows) is seen (**C**). In the DOX + BMSCs + HA group, much milder macrovesicular steatosis in individual hepatocytes (black arrows) is seen (**D**). Magnification 400×.

Normal hepatocyte structure was observed in the control group (Figure 3A). In the DOX group, the hepatic tissues exhibited severe and evident injury with multifocal areas of hepatic macrovascular steatosis in the hepatocytes and mild portal fibrosis with a few leukocytic cell infiltrations (Figure 3B). Mild macrovascular steatosis in a few hepatocytes in the DOX + BMSCs group was detected (Figure 3C). Much milder macrovascular steatosis in individual hepatocytes in the DOX + BMSCs + HA group was examined (Figure 3D).

### 3.6. Effect of HA-Treated BMSCs on the Expression of TGF-β1, SDF1α, and β-Catenin

**Figure 4:**
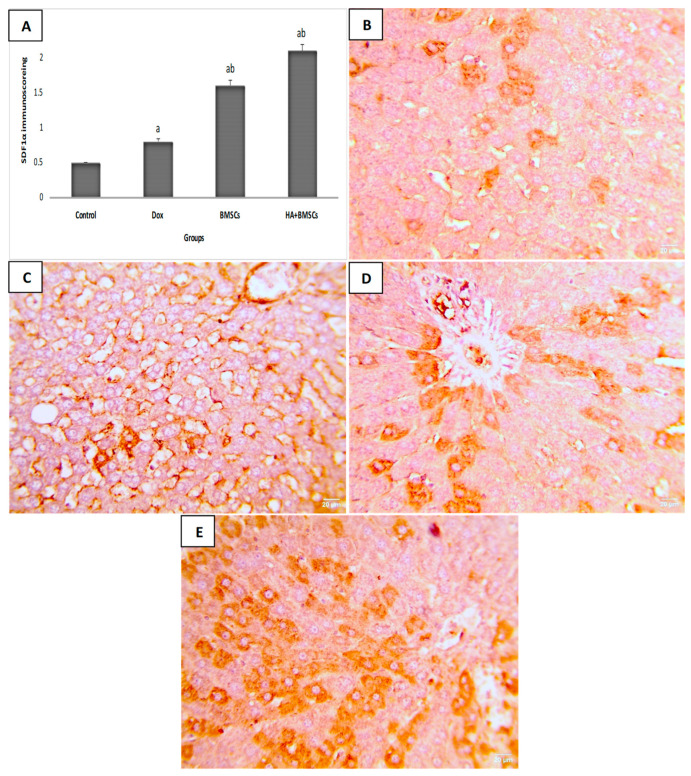
Microscopic pictures of immunostained liver sections against SDF1α. Immune scoring for different studied groups (**A**), low expression in the control group (**B**), mild increase in positive brown expression in hepatocytes in the DOX group (**C**), moderate expression in the DOX + BMSCs group (**D**), and marked expression in the DOX + BMSCs + HA group (**E**). IHC counterstained with Mayer’s hematoxylin. The data are reported as mean ± SD. Significant difference ^a^ vs. control, ^b^ vs. DOX.

**Figure 5:**
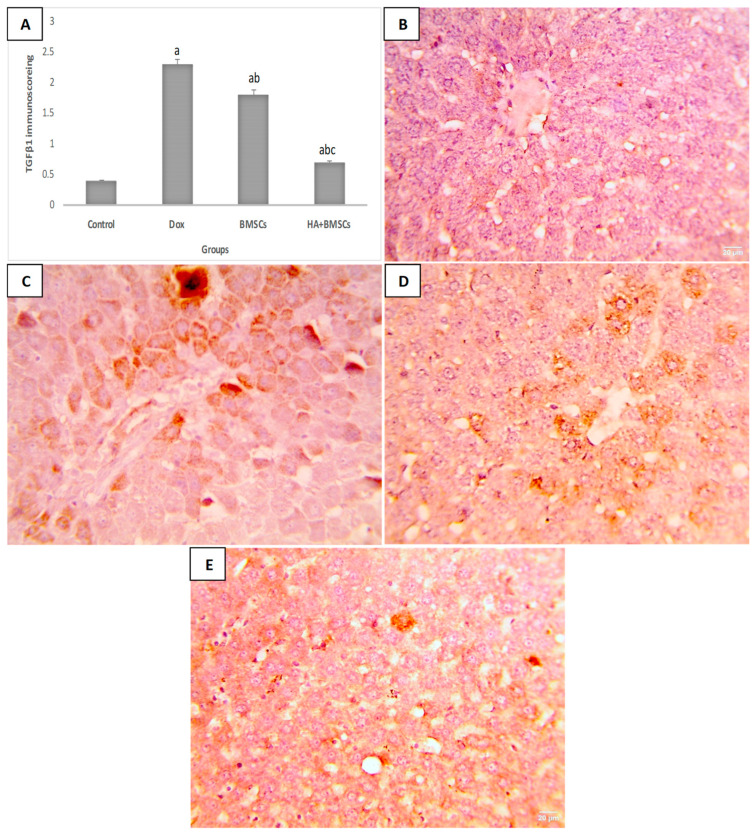
Microscopic pictures of immunostained liver sections against TGFβ1. Immune scoring for different studied groups (**A**), negative expression in the control group (**B**), marked increase in positive brown expression in hepatocytes in the DOX group (**C**), moderate expression in hepatocytes in the DOX + BMSCs group (**D**), and mild expression in hepatocytes in the DOX + BMSCs + HA group (**E**). IHC counterstained with Mayer’s hematoxylin. Magnification 400×. The data are reported as mean ± SD. Significant difference ^a^ vs. control, ^b^ vs. DOX, and ^c^ vs. BMSCs.

**Figure 6:**
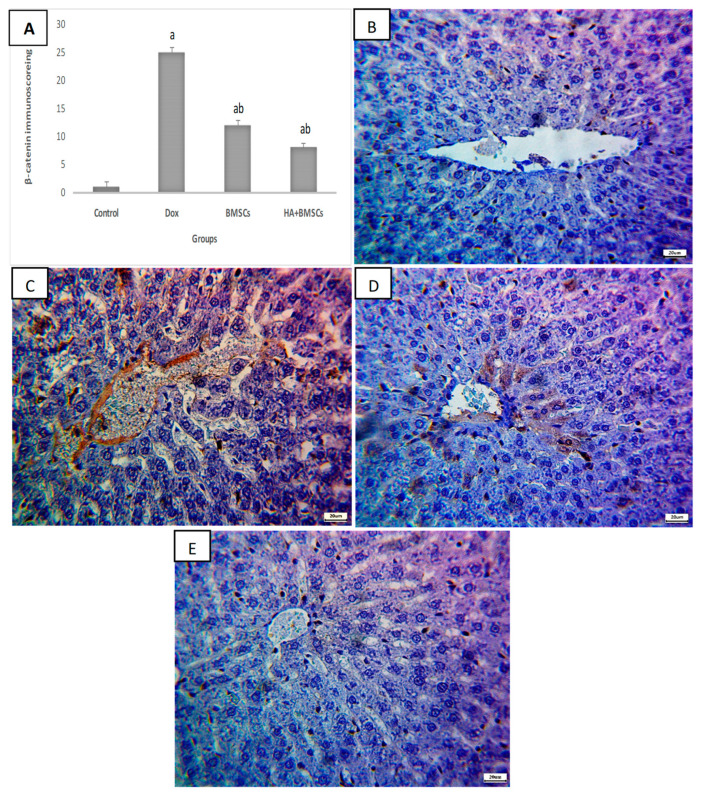
Microscopic pictures of immunostained liver sections against β-catenin. Immune scoring for different studied groups (**A**), negative expression in the control group (**B**), marked expression in hepatocytes in the DOX group (**C**), moderate expression in hepatocytes in the DOX + BMSCs group (**D**), and mild expression in hepatocytes in the DOX + BMSCs + HA group (**E**). Magnification 400×. The data are reported as mean ± SD. Significant difference ^a^ vs. control, ^b^ vs. DOX.

The immunohistochemical scoring of SDF1α expression in liver tissue was significantly increased in the DOX group compared to the control (*p* < 0.01), while in the treated groups, its expression was substantially elevated as compared to the DOX group (*p* < 0.05; Figure 4A). As shown in Figure 4B–E, a mild increase in positive brown expression in hepatocytes was observed in the DOX group. Moderate SDF1α expression in the the DOX + BMSCs group and marked expression in the DOX + BMSCs + HA group was observed. Additionally, the TGF-β1 and β-catenin Immunoscores were remarkedly increased in the DOX group in contrast to the control (*p* < 0.01). The BMSCs and HA + BMSCs groups showed a considerable decrease in TGF-β1 and β-catenin when compared to the DOX group (*p* < 0.01; Figure 5A and Figure 6A). TGF-β1 and β-catenin showed negative expression in the control (Figure 5B and Figure 6B), while marked expression was observed in the DOX group (Figure 5C and Figure 6C). TGF-β1 and β-catenin expression became moderate and mild in the BMSCs and HA + BMSCs groups, respectively (Figure 5D–E and Figure 6D–E).

## 4. Discussion

Doxorubicin (DOX), one of the most effective chemotherapeutics against cancer, is reported to accumulate in patients with liver cancer due to its detoxification capacity. Acute toxicity (short-term model) is caused by a single dose of DOX (usually ranging from 5–30 mg/kg) and can cause liver, cardiac, and renal damage. Chronic DOX toxicity (long-term model) is induced after administering multiple low doses of DOX over a 2–12-week period [21]. Based on previous studies, we used a single dose of DOX to induce acute liver damage in the current study. Furthermore, the single dose of DOX used in this study (20 mg/kg) corresponds to a high dose used clinically with cancer patients [22].

DOX metabolism usually occurs in the liver via microsomal enzymes, and the formation of an immunogenic or toxic intermediate may result in liver damage via free radical production, intercalation into nuclear DNA, decreased transcription and cell proliferation, and injury to the mitochondria and cell membrane structure [4].

BMSC transplantation has opened new insights into the treatment of different diseases; it can differentiate into various cell types, migrate to the injured tissues, and trigger healing processes through paracrine actions that can also reduce tissue damage and promote tissue repair by reducing oxidative stress and limiting uncontrolled liver cell death [12]. Several research articles have indicated the therapeutic effect of mesenchymal stem cells in surviving patients after the administration of chemotherapeutic agents in different models [23,24,25,26]. It is noted that the stem cells transplantations were done after recovery from cancer; therefore, we did not include tumor groups. Rather, we managed the adverse effect results after exposure to chemotherapy. The application of mesenchymal stem cells is debated in the literature, though [27,28] reported that accumulating discoveries regarding stem cell biology have provided new potential approaches to curing cancer patients.

However, methods for boosting BMSCs’ homing to injured hepatic tissues need to be improved. The extracellular matrix and synovial fluids include high concentrations of the natural polysaccharide, hyaluronic acid (HA), that controls various physiological processes, such as inflammation, cell migration, and proliferation, as well as tissue regeneration [29]. This study did not include the HA group due to the adverse effects associated with HA administration. HA has been reported to cause an increased risk of kidney stone disease, particularly the calcium oxalate (CaOx) variety [30], and adversely affects kidney function and morphology, as reported in our previous work [29]. Moreover, HA has been found to correlate with the histological stages of liver fibrosis (F) in chronic liver diseases [31].

The most important results of the present investigation include finding that (i) pretreatment of BMSCs with HA in vitro caused a significant increase in cell viability, (ii) compared to BMSCs alone, HA-pretreated BMSCs led to a considerable improvement in liver function and morphology, and (iii) a hepatoprotective effect was associated with the downregulation of the Wnt/β-catenin pathway, apoptotic markers, and inflammation, besides oxidative stress.

According to the findings of the present in vitro study, pretreatment of BMSCs for 14 days results in an increased proliferation rate and the number of viable cells, indicating that HA treatment enhances cellular viability, proliferation, and increased CD44 gene expression compared to untreated cells, along with increased SDF1α gene expression, suggesting that HA increases cell migration.

Supporting our results, Luo et al. [32] reported that in vitro assays showed that HA supports cell migration and proliferation. In their study, Moreno et al. [33] concluded that the proliferation of BMSCs is stimulated by HA, which is non-toxic to the cells and actually induces an anti-inflammatory phenotype in BMSCs. Moreover, the high expression of CD44 in BMSCs treated with HA is aligned with the findings of Awadalla et al. [29].

The current study evaluated DOX toxicity in terms of liver injury. The results showed considerable liver function deterioration (through a significant increase in AST and ALT with a reduction in albumin levels and histological alterations). Consistent with this, Wali et al. [34] examined the efficacy of naringenin against DOX-induced liver damage and found that there was considerable liver damage in the DOX-treated group via increased liver enzymes (ALT and AST), indicating the toxicity of the administered dose. Similarly, Zhao, Henninger et al., and Afsar et al. discovered elevated serum ALT and AST levels which contributed to DOX-induced liver damage [35,36,37].

HA + BMSCs were presented in the current study as a promising treatment for injured hepatic tissues following the administration of DOX through the normalization of the liver enzymes AST and ALT, as well as a rise in albumin levels that is in line with the results of Shokeir et al. [38], Hussein et al. [39] and Zahran et al. [40].

Consistent with the biochemical parameters, the histological examination of the liver tissue displayed minimal injury with signs of regeneration in the cell-treated groups when compared to the control group. Our findings are supported by others who have shown the efficacy of stem cells in treating liver damage [39,41,42,43,44,45,46,47]. Furthermore, BMSCs preconditioned with HA demonstrated greater therapeutic efficacy than untreated BMSCs, indicating that HA enhances the regenerative capacity of BMSCs and thus increases the efficacy of treatment by promoting cell proliferation, regulation of cell–cell interactions, enrollment in the damaged tissues, and cell–matrix adhesion. These results are consistent with prior research that suggested that HA might improve stem cell treatments [14,48].

Oxidative stress is a key mechanism in DOX-induced liver damage via ROS production at respiratory chain complex I [49]. Our findings demonstrated a significant decline in the antioxidants SOD and GSH with a significant rise in the lipid peroxidation marker MDA in the DOX group, in contrast to the control, suggesting an elevation of oxidative stress during liver injury. Moreover, we observed a significant increase in the antioxidant levels of SOD and GSH, as well as a notable decrease in MDA levels, in the BMSC-treated group, though the efficacy of HA-BMSCs was superior, indicating that reduction of oxidative stress may be a mechanism for the hepatoprotective action of BMSCs pretreated with HA.

In the same context, Jiao et al. [50] and Serag et al. [51] concluded that HA-BMSC clearly diminished the inflammatory response, in addition to promoting angiogenesis. In vitro, MSC-HA improved cell viability, migration, ROS, wound healing, and MDA level assays in addition to the restoration of abnormal expressions of inflammatory genes and antioxidant genes.

One of the main consequential results of the disruption in the balance between the oxidant and antioxidant system is the apoptosis process [52].

DOX induces apoptosis via the production of ROS in addition to an imbalance in the ratio of Bcl-2 to Bax and induction of a lethal mitochondrial impairment [53] that was observed in the DOX group. In the current study, we found considerable downregulation in Bcl2, Nrf-2, and HO-1 expression with significant upregulation in Bax expression in the liver tissues of the DOX group. Additionally, the current findings indicated significant upregulation of BCl2, Nrf-2, and HO-1 genes with significant downregulation of Bax in the cell-treated groups (BMSCs and HA + BMSCs).

In addition, the VEGF gene was significantly upregulated in the liver tissues of the DOX group. Moreover, the BMSC-treated group showed major downregulation of VEGF expression as compared to the control group, while the effect of the HA-BMSC treatment was more powerful than that of BMSCs alone, indicating that this combination provides more anti-apoptotic and anti-inflammatory actions for hepatoprotection against DOX-induced liver injury in rats. These results were in line with those of previous studies that demonstrated enhanced apoptotic and inflammatory processes in liver tissues after prolonged chemotherapy exposure [38,48].

Transforming growth factor (TGF)-β1 has a major effect on the chemo-attracting properties of monocytes and macrophages. During liver injury, TGF-β1 triggers an inflammatory cascade [54]. The TGF-β1 mRNA and protein were remarkably upregulated in the DOX group, based on our investigation. Moreover, the BMSC-treated group exhibited considerable downregulation of TGF-β1 mRNA and protein, which was more pronounced in the HA + BMSCs group than in the BMSCs group, indicating the anti-inflammatory activity of BMSCs in the context of liver damage.

Potent chemo-attractants, the stromal cell-derived factor 1 alpha (SDF1α), and its corresponding chemokine receptor CXCR4, influence the homing and migration of lymphocytes and hematopoietic progenitor cells [55]. In the current study, stem cell groups showed more meaningful upregulation in SDF1α expression than the control and DOX groups. This was also observed by El Nashar et al. [56], Akcora et al. [57], and Du et al. [58]. Moreover, BMSCs preconditioned with HA showed the highest SDF1α level among all treated groups, suggesting the role of HA in enhancing cell migration.

Many biological processes are mediated by the Wnt/β-catenin signaling cascade, such as organogenesis and the pathophysiology of liver homeostatic mechanisms and disorders [59,60,61]. Previous studies have demonstrated the essential role of Wnt/β-catenin signaling in the downregulation of hepatic stellate cell (HSC) activation and an eventual reduction in liver fibrosis [62]. Our results observed that Wnt/β-catenin pathway expression was upregulated significantly in liver tissues after DOX treatment, but significantly downregulated after BMSC and HA + BMSC treatment. Although HA + BMSCs had a greater impact than BMSCs alone, Wnt/β-catenin expression was significantly downregulated in the HA + BMSCs group. These results suggested that the pretreatment of BMSCs with HA might improve liver function and morphology after exposure to chemotherapy via Wnt/β-catenin pathway inhibition.

Moreover, FN1, COL1, and iNOS axis-mediated liver fibrosis were caused by DOX, and our data documented the fibrotic effect of DOX via the upregulation of VEGF, Wnt7b, β-catenin, FN1, Col-1, TGFβ1, iNos, and Bax in the DOX group. This agrees with the results of Anavi et al. [63] that indicated that hepatic fibrosis may be caused by increasing iNOS in mice. However, FN1, COL1, and iNOS were significantly downregulated in the treated groups compared to the DOX group, an effect also reported by Awadalla et al. [29], who found that inflammatory cytokines (iNOS) were upregulated in renal tissues during I/R procedures and found superior therapeutic outcomes for HA + MSCs as compared to MSCs, indicating the anti-apoptotic, anti-inflammatory, and antioxidant effects of MSCs.

Similarly, we looked at inflammation-related genes such as TNF-α and IL-6. We discovered that DOX treatment increased the expression of these genes. These findings were consistent with previous research [22]. Nonetheless, as previously mentioned by Chen et al., MSC treatment ameliorated these changes in TNF-α and IL-6 gene expression [64]. Furthermore, BMSCs pretreated with HA manifested the lowest expressions of TNF-α and IL-6, suggesting its anti-inflammatory role, as reported by Kim et al. [65].

## 5. Conclusions

The pretreatment of BMSCs with HA in vitro enhances the ability of the stem cells to proliferate and migrate via the upregulation of cell surface molecules and anti-inflammatory cytokines. Administration of these cells in the liver has a more powerful hepatoprotective effect than administration of BMSCs alone. This hepatoprotective effect is possible because of the upregulation of the stem cell trafficking marker (SDF1α) and downregulation of the Wnt/β-catenin signaling pathway, apoptosis, and oxidative stress.

## Figures and Tables

**Table 1:** PCR primer sequence.

Common Name	Sequence (5′-3′)	Accession No.
Wnt7b	F: 5′-CCATCATCGTGATCGGGGAG-3′ R: 5′-TCCAGTTTCATGCGGTCCTC-3′	NM_001009695.1
iNOS	F: 5′-AGTCAACTACAAGCCCCACG-3′ R- 5′-GCAGCTTGTCCAGGGATTCT-3′	NM_012611.3
BCL2	F: 5′-GGTGAACTGGGGGAGGATTG-3′ R: 5′-GCATGCTGGGGCCATATAGT-3′	NM_016993.1
VEGF	F: 5′-ACGAAAGCGCAAGAAATCCC-3′ R: 5′-TTAACTCAAGCTGCCTCGCC-3′	NM_031836.3
Bax	F: 5′-GGCGATGAACTGGACAACAA-3′ R: 5′-CAAAGTAGAAAAGGGCAACC-3′	NM_017059.2
SDF1*a*	F: 5′-GAGCCATGTCGCCAGAGCCAAC R: 5′-CACACCTCTCACATCTTGAGCCTCT	NM_001033882.1
Collagen-1	F: 5′-ACGTCCTGGTGAAGTTGGTC-3′ R: 5′-CAGGGAAGCCTCTTTCTCCT-3′	NM_ 29,393
TGF-β1	F: 5′-CACTCCCGTGGCTTCTAGTG-3′ R: 5′-GGAC TGGCGAGCCTTAGTTT-3′	NM_021578.2
β-catenin	F: 5′-ACAGCACCTTCAGCACTCT-3′ R: 5′-AAGTTCTTGGCTATTACGACA-3′	NM_053357.2
Nrf2	F: 5′-ATTGCTGTCCATCTCTGTCAG-3′ R: 5′-GCTATTTTCCATTCCCGAGTTAC-3′	NM_001399173.1
HO-1	F: 5′-TGCTTGTTTCGCTCTATCTCC-3′ R: 5′-CTTTCAGAAGGGTCAGGTGTC-3′	NM_012580.2
Fibronectin	F: 5′-GTGGCTGCCTTCAACTTCTC-3′ R: 5′-GTGGGTTGCAAACCTTCAAT-3	NM_U82612.1
CD44	F: 5′-TGGCACAGCAGCAGATC-3′ R: 5′-CTGCACAGATAGCGTTGG-3′	NM_012924.3
IL_6	F: 5′-CGAGCCCACCAGGAACGAAAGTC-3′ R: 5′-CTGGCTGGAAGTCTCTTGCGGAG-3′	NM_012589.2
TNF-α	F: 5′-TTC GGA ACT CAC TGG ATC CC-3′ R: 5′-CGGA ACA GTC TGG GAA GCT CT-3′	NM_012675.3
GAPDH	F: 5′-AGACAGCCGCATCTTCTTGT-3′ R: 5′-TTCCCATTCTCAGCCTTGAC-3′	NM_017008.4

**Table 2:** The mean value of liver enzymes and oxidative markers in the studied groups.

	Control	DOX	BMSCs	BMSCs + HA
ALT (U/L)	49 ± 3.4	137.83 ± 6.43 ^a^	98.67 ± 10.24 ^ab^	77.5 ± 15.50 ^abc^
AST (U/L)	104.16 ± 15.7	261.16 ± 52.02 ^a^	149.66 ± 49.2 ^b^	117.33 ± 20.64 ^b^
Alb (g/dL)	3.51 ± 0.55	1.98 ± 0.25 ^a^	2.48 ± 0.68 ^a^	3.12 ± 0.41 ^b^
CAT (U/gm)	8.1 ± 1.96	1.6 ± 0.42 ^a^	5.25 ± 0.82 ^ab^	6.4 ± 1.4 ^b^
GSH (U/gm)	10.21 ± 3.28	3.16 ± 0.95 ^a^	7.5 ± 1.4 ^b^	9.2 ± 1.59 ^b^
SOD (U/gm)	289.41 ± 37.1	115.26 ± 32.53 ^a^	220.27 ± 41.9 ^ab^	273.48 ± 23.05 ^b^
MDA (nmol/gm)	77.38 ± 10.89	168.44 ± 15.77 ^a^	113.37 ± 14.43 ^ab^	88.83 ± 14.47 ^bc^

The data are reported as mean ± SD. Significant difference ^a^ vs. control, ^b^ vs. DOX, and ^c^ vs. BMSCs. The reference ranges of AST, ALT, and Albumin are 50–150 IU/L, 10–40 IU/L, and 3.8–4.8 g/dL, respectively [20].

## Data Availability

All raw data are available on request.
